# Negotiating assistive technologies and AI in inclusive education: professional agency in neurodivergent contexts

**DOI:** 10.3389/frcha.2026.1820276

**Published:** 2026-04-13

**Authors:** Arianna Marras, Angela Pasqualotto

**Affiliations:** 1 Department of Humanities, Languages and Cultural Heritage, University of Cagliari, Cagliari, Italy; 2 Department of Education and Learning, University of Applied Sciences and Arts of Southern Switzerland, Locarno, Switzerland

**Keywords:** artificial intelligence, assistive technologies, inclusive education, neurodevelopmental disorders, teachers

## Abstract

**Introduction:**

Digital assistive technologies and AI-based systems are increasingly introduced in inclusive classrooms serving students with neurodevelopmental differences. Yet their implementation depends on how teachers interpret, evaluate, and regulate these tools within professional practice. This study examines teachers’ knowledge, perceptions, and professional reasoning regarding traditional assistive technologies and emerging AI-based systems.

**Methods:**

A qualitative study was conducted using seven semi-structured focus groups with 46 elementary and middle school teachers (classroom and specialized). Data were analyzed through reflexive thematic analysis using a hybrid inductive-deductive approach, with attention to interactional dynamics.

**Results:**

Five interrelated themes emerged. Teachers showed heterogeneous and sometimes ambiguous conceptualizations of assistive and AI-based systems. Traditional tools were largely framed as individualized accommodations, whereas AI-based systems generated uncertainty and negotiation. Adoption was described as conditional upon pedagogical fit, professional control, institutional feasibility, and ethical accountability. Concerns centered on data governance, responsibility for decision-making, and equity risks.

**Discussion:**

Implementation operated as a process of conditional integration: technologies gained legitimacy only when aligned with inclusive commitments, professional agency, and governance clarity. Sustainable integration depends less on technological expansion than on teachers’ informed professional judgment.

## 1 Introduction

### 1.1 Digital assistive technologies in inclusive education

In recent years, digital Assistive Technologies (AT) have become increasingly embedded within educational systems, paralleling the broader shift toward more inclusion-oriented school models. Within this perspective, inclusive education refers to the intentional design of educational environments to ensure that all students can access and participate in learning experiences, in accordance with principles of equity and respect for diversity (1). AT are positioned squarely within this framework, as the literature consistently recognizes their role in supporting students with disabilities and special educational needs, reducing barriers and enabling fuller participation in learning and school life (2). They have also been increasingly incorporated into interventions targeting neurodevelopmental differences, with applications spanning communication, executive functioning, and adaptive learning supports (3, 4).

Traditionally, AT have been conceptualized as individualized and compensatory tools, selected in response to specific functional needs and aimed at enhancing students’ autonomy, independence, and performance in academic tasks and classroom interactions (5, 6). Within this understanding, technology does not operate autonomously; rather, teacher mediation remains central. “E-inclusion” emerges as a set of professional decisions through which technologies are meaningfully integrated into instructional activities to provide alternative means of access, expression, and participation, ensuring alignment among educational goals, learner variability, and available tools (7). Consistent with approaches that emphasize active participation and barrier removal, AT can therefore be understood not merely as aids “for someone,” but as resources that may contribute to the development of more flexible and accessible learning environments (8).

Despite this established role, research on technologies for inclusion has predominantly concentrated on issues of effectiveness, usability, and tool design. Comparatively less attention has been devoted to the classroom-level processes through which teachers interpret, negotiate, and integrate these technologies within everyday pedagogical practice (9). Recent reviews explicitly call for more implementation-oriented research capable of examining how technological tools are translated into situated instructional decisions. In particular, systematic reviews highlight persistent challenges related to teacher preparation, pedagogical integration competencies, and the organizational and infrastructural conditions that make implementation sustainable - or, conversely, fragile (10, 11).

As emphasized by Karagianni and Drigas (12), e-inclusion cannot be reduced to digital expansion alone, but depends on professional judgments aimed at integrating technologies in ways that both compensate for functional difficulties and enhance students’ strengths. Similarly, Hoogerwerf et al. (1) underline that the effectiveness of AT ultimately hinges on learners’ capacity to use them autonomously and reflectively. Translating technological innovation into concrete outcomes of equity and participation therefore depends not only on functional adequacy, but on its alignment with contextual, pedagogical, and learner-specific conditions.

### 1.2 From assistive technologies to AI-based systems in inclusive education

Alongside the progressive institutionalization of digital assistive technologies, recent years have witnessed the accelerated integration of Artificial Intelligence (AI)–based systems within educational contexts, with significant implications for inclusive education. To clarify the discontinuity between different forms of technological mediation, it is essential to distinguish between: (a) AT, typically understood as supports oriented toward specific tasks and skills - such as access to content, reading, writing, or communication - and (b) AI systems, defined as technologies that process data and generate outputs (e.g., recommendations, feedback, adaptive pathways, or original content) through algorithmic mediation operating at the level of instructional processes (13, 14).

This evolution represents more than incremental technological refinement. While traditional AT primarily extend functional access within teacher-guided frameworks, AI systems introduce adaptive, inferential, and generative capacities that may co-configure the learning environment itself. Through data-driven personalization, automated feedback, and generative models, AI technologies can influence planning and assessment, partially redistributing instructional decision-making (15–17). Empirical evidence suggests that such systems can scale formative feedback efficiently (18), yet their educational validity remains contingent upon sustained professional oversight.

At the institutional level, this shift has been acknowledged in international policy frameworks, which position AI not merely as a supportive instrument but as a strategic lever for systemic educational transformation, while simultaneously emphasizing the need to safeguard human agency and social learning from excessive algorithmic automation (19). As AI systems increasingly mediate recommendations and learning trajectories, issues of transparency, explainability, and accountability become central (20, 21). Integration therefore depends not only on perceived effectiveness, but on governance structures capable of clarifying data practices, delineating professional responsibility, and anticipating distributive consequences (10).

In this sense, AI does not simply expand the technological repertoire available to teachers; it reconfigures the teacher–student–technology relationship and reshapes the distribution of control and responsibility within inclusive educational systems (13, 14). Understanding how teachers interpret and negotiate this redistribution of control becomes therefore central to examining the implementation of AI within inclusive educational practice.

### 1.3 Teachers’ professional agency in inclusive contexts

#### 1.3.1 Agency and the integration of assistive technologies

In light of the reconfiguration of technological mediation outlined above, teachers occupy a central role as mediators and gatekeepers of technological adoption within inclusive educational systems. Their professional judgments shape how assistive technologies are selected, adapted, and sustained in practice, determining whether they function as instruments of participation or remain marginal add-ons. Research consistently shows that teachers’ perceptions, technological competence, and sense of self-efficacy significantly influence both the depth and continuity of AT integration (22), while knowledge of available tools, training opportunities, and institutional support are closely associated with perceived usefulness and sustainable implementation (23). Within this framework, teacher agency encompasses the responsibility to ensure equitable access, safeguard student autonomy, and exercise ethical–pedagogical judgment. AT are not neutral interventions; their effectiveness depends on situated professional decisions that align technological affordances with curricular goals, learner variability, and contextual constraints. Accordingly, limited knowledge and confidence constitute significant barriers to systematic integration (24), whereas targeted professional development and sustained institutional support foster more intentional and durable practices (23, 25).

#### 1.3.2 Agency and the integration of AI-based systems

AI-based systems intensify and reshape professional agency. As algorithmic systems increasingly mediate recommendations, feedback, and adaptive pathways, questions of accountability and the boundaries of pedagogical control become central to teachers’ professional practice. In this context, agency no longer concerns only the selection and adaptation of tools, but also the critical evaluation and governance of algorithmically generated outputs. From a socio-technical perspective, adoption emerges through the interaction of professional beliefs, organizational cultures, regulatory frameworks, and material infrastructures, within which responsibilities and decision boundaries are negotiated (26). Empirical findings indicate that teachers tend to adopt a cautiously positive stance toward AI: while it is often perceived as potentially useful for enhancing efficiency and personalization, concerns persist regarding operational control, ease of use, and its qualitative impact on teaching practice (27). Notably, although perceived usefulness is frequently rated highly, confidence in the system's “governability” varies considerably, underscoring the need for structured professional development and clear institutional guidance prior to full-scale integration.

### 1.4 Neurodivergent contexts as high-stakes implementation environments

Neurodivergent contexts render decisions concerning the adoption and regulation of technologies particularly consequential, as they are characterized by substantial intra- and inter-individual heterogeneity, complex constellations of cognitive, motor, and socio-communicative differences, and dynamically evolving support needs (4, 28). Under such conditions, choices regarding the selection, modes of use, and supervision of technologies may directly affect students’ autonomy, participation, and educational trajectories, amplifying the professional responsibility associated with technological integration. Historically, AT in educational and rehabilitative settings have been grounded in individualized accommodation, closely linked to diagnostic categories and specific needs (e.g., augmentative communication, motor or cognitive supports) (29). Such approaches have often prioritized targeted functions, sometimes at the expense of broader considerations of activity and participation (29, 30).

AI-based systems introduce a potential discontinuity within this framework. Through adaptive and generative logics, AI may blur the boundaries between individualized accommodation, universal design approaches, and automated decision-support systems, influencing not only access to specific tasks but the configuration of the learning experience itself (10, 15). Algorithmic inference intersects with pre-existing classificatory systems (e.g., diagnostic labels) potentially amplifying categorization effects and stabilizing representations of students’ abilities. In settings marked by heightened vulnerability and variability, the quality of algorithmic inferences and their alignment with professional judgment become especially critical. Even minor misclassifications or insufficiently contextualized recommendations may have disproportionate consequences for students’ autonomy, perceived competence, and trajectories of inclusion.

Data governance concerns are particularly salient in neurodivergent contexts due to the sensitivity of student information and structural inequalities. Recent analyses of digital tools targeting neurodevelopmental needs indicate that, despite widespread availability and generally positive usability perceptions, the empirical base supporting their effectiveness remains limited or heterogeneous (13, 30). This evidentiary uncertainty further amplifies the role of professional discretion concerning the enactment of technological supports. These characteristics heighten the stakes of how technologies are enacted in practice, making teachers’ interpretative and regulatory role central to responsible integration.

### 1.5 Implementation and the underexplored interpretative layer

While research has extensively examined effectiveness, usability, and adoption factors, less attention has been devoted to how teachers interpret and integrate these technologies into everyday practice (24, 27). Relatively little is known about how educators distinguish between assistive and AI-based systems, negotiate boundaries between individualized accommodation and universal design, or align adaptive and generative tools with inclusive commitments. In neurodivergent contexts, this interpretative dimension is particularly consequential, as integration entails decisions affecting student autonomy, pedagogical coherence, and institutional sustainability.

Socio-technical perspectives suggest that technologies are reshaped through professional cultures and organizational conditions (26), yet this process of translation remains underexplored. Addressing this gap requires qualitative inquiry into teachers’ knowledge, perceptions, and professional reasoning, as teachers function as the primary implementation interface between technological innovation and inclusive educational practice.

### 1.6 The present study

Despite the growing body of research on assistive technologies and AI in education, a significant gap remains in understanding how teachers interpret, negotiate, and regulate these technologies within everyday inclusive classroom practice, particularly in contexts involving students with neurodevelopmental differences.

The aim of the present study is to explore teachers’ perceptions and professional reasoning regarding the use of digital assistive technologies and emerging AI-based systems in inclusive educational settings. To address this objective, we conducted semi-structured focus groups with elementary and middle school teachers working in inclusive contexts serving students with neurodevelopmental differences.

Specifically, the study addresses the following research questions:

1. How do teachers conceptualize the relationship between traditional assistive technologies and emerging AI-based tools?
2. How do teachers evaluate the pedagogical usefulness and contextual fit of these technologies in classroom practice?
3. What individual and organizational factors influence teachers’ adoption of these technologies?
4. What ethical and governance-related concerns do teachers identify in relation to AI-enabled educational tools?
5. Under what conditions do teachers consider the integration of these technologies to be responsible and sustainable in inclusive classrooms?

Particular attention was paid to potential variations associated with professional role (classroom vs. specialized teachers) and school level. By centering teachers’ professional reasoning in high-stakes inclusive contexts, this study contributes to a deeper understanding of how adaptive and data-driven technologies are interpreted, negotiated, and embedded within everyday educational decision-making.

## 2 Methods

### 2.1 Study design

A qualitative design based on semi-structured focus groups was employed to explore teachers’ knowledge, perceptions, and professional reasoning regarding digital assistive technologies, including AI-enabled tools, in inclusive educational contexts. Such an approach was appropriate to examine how meanings around neurodiversity, participation, and technological support are constructed and negotiated within everyday school practice.

Focus groups were selected because they generate interactional data, enabling participants to respond to, challenge, and elaborate upon one another's perspectives (31, 32). This method is particularly suited to examining issues of inclusion, professional responsibility, and ethical technology use, which are shaped through shared institutional cultures and collegial discourse rather than individual attitudes alone. Comparable qualitative approaches have been used to investigate teachers’ perspectives on assistive technologies within educational systems (26).

The research was grounded in a constructivist epistemological framework, recognizing that understandings of innovation, accessibility, and ethical responsibility are socially situated and embedded within professional and institutional contexts. Reporting followed the Consolidated Criteria for Reporting Qualitative Research [COREQ; (33)].

### 2.2 Participants and recruitment

A total of 46 teachers participated in the study (31 females, 15 males; mean age=34 years, range=25–46). Participants were recruited from the Italian-speaking regions of Switzerland and Northern Italy and were working in inclusive educational settings serving students with neurodevelopmental differences.

Twenty participants were elementary school teachers and 26 were middle school teachers. Twenty-two participants were classroom teachers and 24 held specialized or support roles. Thirty-five participants reported prior experience with assistive technologies, and 12 reported prior experience with AI-based educational tools. Participant characteristics are summarized in Table 1.

**Table 1:** Participants’ characteristics.

Variable	*n*	%
Gender		
Female	31	67%
Age (years)		
Mean (range)	34 (25–46)	—
School level		
Elementary	20	43%
Middle school	26	57%
Role		
Classroom teacher	22	48%
Specialized/support teacher	24	52%
Prior experience with AT		
Yes	35	76%
Prior experience with AI-based tools		
Yes	12	26%

A purposive sampling strategy was employed to ensure variation in professional role and school level relevant to inclusive practice (34). Seven focus groups were conducted, each including 6–8 participants. Groups were composed homogeneously by school level (elementary or middle school) to facilitate discussion grounded in shared pedagogical contexts (31, 32). Classroom and support teachers were included within the same groups to encourage dialogue across complementary professional perspectives. Participation was voluntary.

### 2.3 Data collection

Data collection took place between March and October 2025. Focus groups were conducted in Italian. Transcripts were analyzed in the original language, and excerpts included in the manuscript were translated into English by the research team.

A semi-structured discussion guide (see Supplementary Table S1) was developed to structure the conversation while preserving flexibility. Questions progressed from broad reflections on inclusive practices to more focused prompts concerning experiences with assistive technologies and AI-enabled tools. Open-ended questions and follow-up probes were used to elicit examples, clarify meanings, and explore points of agreement or divergence within the group.

Each focus group lasted approximately 60–90 min and was moderated by a clinical psychologist with expertise in neurodevelopmental disorders and inclusive education research. Participants were informed of the moderator's professional background prior to the discussion. The moderator had no supervisory relationship with participants and facilitated balanced participation without directing the substance of responses. Sessions were audio-recorded and transcribed verbatim.

Ethical approval was obtained from the relevant institutional ethics committee prior to data collection. Participants provided written informed consent and were informed of their right to withdraw at any time. Given the group-based format, participants were reminded that confidentiality could not be fully guaranteed within the group and were asked to respect the privacy of other participants. Transcripts were anonymized and securely stored in compliance with applicable data protection regulations.

Data collection continued until thematic sufficiency was reached, defined as the point at which additional focus groups did not substantially extend or modify the emerging thematic structure (35).

### 2.4 Analytic approach

Data were analyzed using reflexive thematic analysis (36, 37). This approach was selected to identify patterned meanings across the dataset while acknowledging the interpretative role of the research team.

In analyzing focus group data, attention was paid not only to the content of individual statements but also to interactional features, including moments of consensus, disagreement, amplification, and hesitation. Such dynamics were treated as analytically meaningful, as they illuminate how professional positions are collectively negotiated within group settings (31).

The analysis proceeded through iterative phases of familiarization, initial coding, theme development, review, and refinement. Following transcription, each focus group was read repeatedly to support immersion in the dataset as a whole and sensitivity to interactional dynamics within and across groups. Initial coding was conducted line-by-line on meaning units relevant to teachers’ conceptualizations, experiences, evaluative judgments, and concerns regarding assistive technologies and AI-based tools. Codes were then compared across transcripts and progressively clustered into candidate categories capturing recurrent interpretative patterns. These categories were subsequently developed into broader themes through iterative movement between coded extracts and the full dataset. Coding was conducted manually using structured coding matrices. Coding operated at both semantic and latent levels, allowing examination of explicit accounts of technology use as well as underlying assumptions about neurodiversity, inclusion, equity, and professional agency.

A hybrid inductive–deductive strategy was adopted. Initial coding was data-driven to remain grounded in teachers’ narratives. Interpretation was subsequently informed by sensitizing concepts derived from inclusive education and implementation scholarship, including teachers’ conceptual understanding of assistive technologies, perceived pedagogical fit, determinants of adoption at individual and organizational levels, perceptions of ethical and equity-related risks, and conditions for implementation feasibility (e.g., training, institutional support, integration into practice) (38–40). These dimensions functioned as interpretive lenses rather than predefined coding categories.

Analytic decisions were discussed collaboratively within the research team, and an audit trail documenting coding processes and theme refinement was maintained throughout. Reflexive consideration of researchers’ positionality in relation to inclusive education and digital innovation informed interpretative decisions. Theme refinement involved repeated comparison between candidate themes, coded extracts, and the dataset as a whole in order to assess coherence, distinctiveness, and explanatory fit. During this process, some preliminary categories were collapsed, re-specified, or repositioned when they were found to overlap conceptually or to function better as subthemes rather than standalone themes. The final thematic structure was retained when it provided a coherent account of patterned meaning across groups without obscuring areas of tension, ambiguity, or variation. An overview of illustrative open codes and their development into subthemes and final themes is provided in Supplementary Table S2.

## 3 Results

Analysis generated five interrelated themes reflecting teachers’ conceptualizations, adoption logic, structural constraints, ethical considerations, and conditions for responsible integration of digital assistive technologies and AI-based systems in inclusive educational contexts.

### 3.1 Theme 1: ambiguity and divergent conceptualizations of assistive technologies and AI-based systems

Across focus groups, teachers demonstrated heterogeneous and sometimes fragmented understandings of what constitutes digital assistive technology, particularly when AI–based tools were introduced. While traditional assistive technologies were generally recognized and concretely described, AI systems were framed in more abstract terms and often associated with uncertainty.

Assistive technology was frequently defined in individualized and compensatory ways, closely linked to students with formally recognized neurodevelopmental difficulties. As one participant stated, “Assistive technology, for me, is something you give to a student who has a certification … it's something specific for that child” (P8, Elementary). This framing reflects a persistent association between assistive technology and individualized remediation rather than broader inclusive or universal design approaches.

In some groups, this individualized framing was expanded through interaction. Specialized teachers occasionally articulated a more inclusive perspective, suggesting that tools such as text-to-speech software could benefit the entire classroom. One participant explained, “Sometimes it's easier if everyone uses the tool. That way it's not just for one student” (P12, Specialized, Middle). These exchanges indicate that assistive technologies were sometimes repositioned from individualized accommodations to supports for collective participation.

When conversation shifted toward AI-based tools, conceptual ambiguity intensified. Participants frequently attempted to define AI collaboratively, sometimes expressing uncertainty and seeking clarification from peers. As one participant explained:

I think we use the word AI very easily now, but I am not sure what it really means in practice. Is it just something that adapts automatically? Or does it mean that the system is making decisions on its own? Sometimes I feel we say AI, but we are just talking about normal digital tools. (P21, Middle)

In several groups, similar reflections prompted brief exchanges in which participants compared examples and questioned whether specific applications truly qualified as artificial intelligence. These interactional moments suggest that AI remains an evolving and partially stabilized concept within professional discourse.

Across discussions, assistive technologies were typically framed as tools supporting student autonomy, for example by enabling reading, writing, or organizational independence. AI systems, by contrast, were more frequently associated with personalization and adaptive decision-making. This distinction shaped how teachers positioned the two categories. Assistive technologies were perceived as concrete and embedded within established support practices, whereas AI systems were seen as more systemic and less transparent.

AI was more readily accepted when conceptualized as a professional support for teachers, such as generating instructional materials or adapting texts during lesson preparation, than when envisioned as directly guiding student learning. Several participants described experimenting with AI for planning while expressing hesitation about student-facing use. As one teacher noted, “For planning, it can be helpful … but I'm not sure I would let it guide the students directly” (P27, Middle). This distinction suggests differing thresholds of perceived pedagogical and ethical risk.

A further pattern involved assumptions that digital tools are inherently inclusive. Some participants initially associated technology with increased accessibility. However, these claims were frequently moderated within group discussions, with others emphasizing that effectiveness depends on informed pedagogical use. As one teacher observed, “Technology helps everyone, but only if you really know how to use it properly” (P4, Elementary).

In sum, divergent conceptualizations differentiated traditional assistive technologies from AI-based systems. Assistive technologies were primarily framed as autonomy-support tools embedded in established inclusive practices, particularly for students with neurodevelopmental differences. AI systems were associated with personalization potential and professional support functions but accompanied by greater conceptual uncertainty. These foundational distinctions shaped subsequent discussions of adoption, feasibility, and risk.

### 3.2 Theme 2: conditional acceptance and contextualized adoption

Openness toward digital and AI-based technologies was consistently framed as conditional. Adoption depended on perceived pedagogical value, alignment with curricular demands, and contextual feasibility rather than on enthusiasm for innovation alone.

Technologies were viewed positively when they supported clearly articulated instructional goals. Tools that enhanced comprehension, scaffolded writing, or facilitated differentiated instruction were described as beneficial, particularly for students with diverse learning profiles. As one participant noted, “If it really helps the student understand better, then of course I would use it” (P14, Elementary). In such cases, assistive technologies were linked to increased autonomy and participation.

AI was also associated with potential benefits, particularly in relation to personalization and material adaptation. Some teachers described AI systems as capable of adjusting text complexity or generating differentiated exercises. However, in several discussions, positive statements about personalization were followed by careful consideration of appropriateness and control.

Acceptance varied depending on the function assigned to AI. When framed as a planning aid that supported lesson preparation or content generation, AI was often perceived as time-saving and professionally useful. One participant explained, “For preparing lessons, it can save time and give ideas. But I am more careful when it comes to students using it directly” (P22, Middle). In contrast, direct use with students, particularly younger learners or those with neurodevelopmental vulnerabilities, elicited more cautious responses.

Role-based nuances emerged in adoption logic. Specialized teachers more frequently emphasized flexible integration of assistive tools within inclusive classrooms, including their potential use by entire groups. Classroom teachers, by contrast, often foregrounded curriculum alignment and workload considerations. As one classroom teacher observed, “It has to fit with what we are required to teach. Otherwise, it becomes an extra burden” (P18, Middle). These differences indicate that professional role shapes how feasibility and value are evaluated.

Expectations regarding empirical validation also influenced acceptance. Several participants expressed a desire for evidence of effectiveness in real classroom contexts. At the same time, collaborative school cultures were described as facilitating experimentation. Adoption was therefore embedded within professional identity and institutional environment rather than treated as an individual preference.

### 3.3 Theme 3: structural and organizational frictions in implementation

Despite conditional openness, implementation remained strongly mediated by structural and organizational conditions. Integration depended not only on attitudes but also on training, infrastructure, and institutional coherence.

Limited professional development emerged as a central concern. Participants reported insufficient structured training on evaluating and integrating digital tools in inclusive practice. One teacher stated, “We are expected to use these tools, but no one really trains us properly” (P11, Specialized Elementary). In several groups, similar accounts were exchanged, reinforcing the perception of a gap between expectations and support.

Time constraints further restricted sustained experimentation. Teachers described balancing curricular demands with administrative responsibilities, leaving limited space for innovation. As one middle school teacher noted, “There is never enough time to experiment. We barely manage the regular program” (P26, Middle). These comments were often met with agreement from other participants, underscoring shared pressures.

Infrastructure reliability was also linked to pedagogical trust. Participants referred to inconsistent connectivity and limited devices as barriers to continuity. “Sometimes the idea is good, but if the system doesn't work reliably, we stop using it” (P19, Elementary).

Professional role influenced how constraints were interpreted. Specialized teachers emphasized coordination between classroom and support staff when implementing assistive tools, particularly when used inclusively across groups. Classroom teachers more frequently highlighted tensions between innovation and accountability requirements.

Administrative complexity constituted an additional layer of friction. Documentation procedures and approval processes were described as burdensome. “It's not just using the tool. There is paperwork, permissions, coordination. It becomes heavy” (P30, Middle).

Importantly, these constraints were not framed as insurmountable. Many participants expressed interest in innovation but emphasized the need for systemic alignment, protected time, and institutional leadership. Implementation was therefore conceptualized as an organizational process rather than an individual choice.

### 3.4 Theme 4: ethical tensions and professional responsibility in the context of AI

Ethical reflection was most pronounced when discussing AI-based systems. While traditional assistive technologies were rarely described as ethically problematic, AI tools elicited more sustained discussion regarding responsibility, transparency, and systemic implications.

Data privacy concerns were recurrent. Participants expressed uncertainty about data governance when using AI-enabled platforms. “We enter sensitive information about students, but we don't really know where it goes or who can access it” (P17, Middle). Such concerns were less frequently associated with conventional assistive tools.

Algorithmic opacity was also described as professionally challenging. Teachers articulated discomfort with explaining AI-generated recommendations to parents or students. “If the system suggests something, I don't always understand why” (P9, Elementary). In group discussions, concerns about opacity were sometimes balanced by comments emphasizing the importance of teacher oversight. One participant elaborated on this tension in greater detail:

I can see that it might help personalize things for students who struggle, and that is important. But if I cannot explain how it works, especially to parents, I feel uncomfortable. I am responsible for what happens in my classroom. If the system makes a recommendation, I need to understand it and decide whether it makes sense for that student. Otherwise, I am accountable for something I do not really control. (P24, Specialized, Middle)

This reflection was met with agreement from other participants, who emphasized the importance of maintaining professional oversight.

Dependency risks were discussed primarily in relation to AI-driven automation. Some participants worried that excessive reliance might reduce independent skill development. At the same time, others argued that careful monitoring could mitigate such risks.

Ethical reflection was not exclusively framed in negative terms. Several teachers acknowledged that AI-driven personalization, when accompanied by professional oversight, could enhance access for students with neurodevelopmental differences by adapting materials to individual learning profiles. As one participant noted, “If it adjusts the level for the student and we monitor it, it could really make things more accessible” (P31, Elementary). In these exchanges, ethical acceptability was closely linked to retained teacher judgment and contextual control.

At the same time, concerns about inequality amplification extended beyond individual classrooms. AI-based tools were perceived as potentially widening systemic gaps if access to advanced technologies varied across schools or regions. Participants emphasized that without coordinated policy and equitable resource allocation, digital innovation could reproduce or intensify existing educational disparities.

### 3.5 Theme 5: preconditions for responsible and inclusive integration

Discussions culminated in articulating conditions under which digital and AI-based technologies could align with inclusive education principles. Teachers described a model of conditional innovation grounded in professional agency and institutional support.

Sustained professional development was identified as foundational. Participants emphasized the need to connect technical knowledge with pedagogical reasoning. “We don't just need to know how the tool works. We need to understand when and why to use it” (P6, Elementary).

Clear institutional guidelines were viewed as enabling rather than restrictive, particularly regarding AI governance. Collaborative decision-making across roles was also emphasized.

Human oversight was consistently described as indispensable. Even when acknowledging personalization potential, participants stressed that final decisions should remain with teachers.

Finally, teachers returned to the relational core of inclusion. Technology was acceptable when it enhanced participation and autonomy without displacing human interaction. Responsible integration was thus conceptualized as a systemic and ethical process embedded within inclusive educational commitments.

### 3.6 Theme 6: preconditions for responsible and inclusive integration

**Figure 1:**
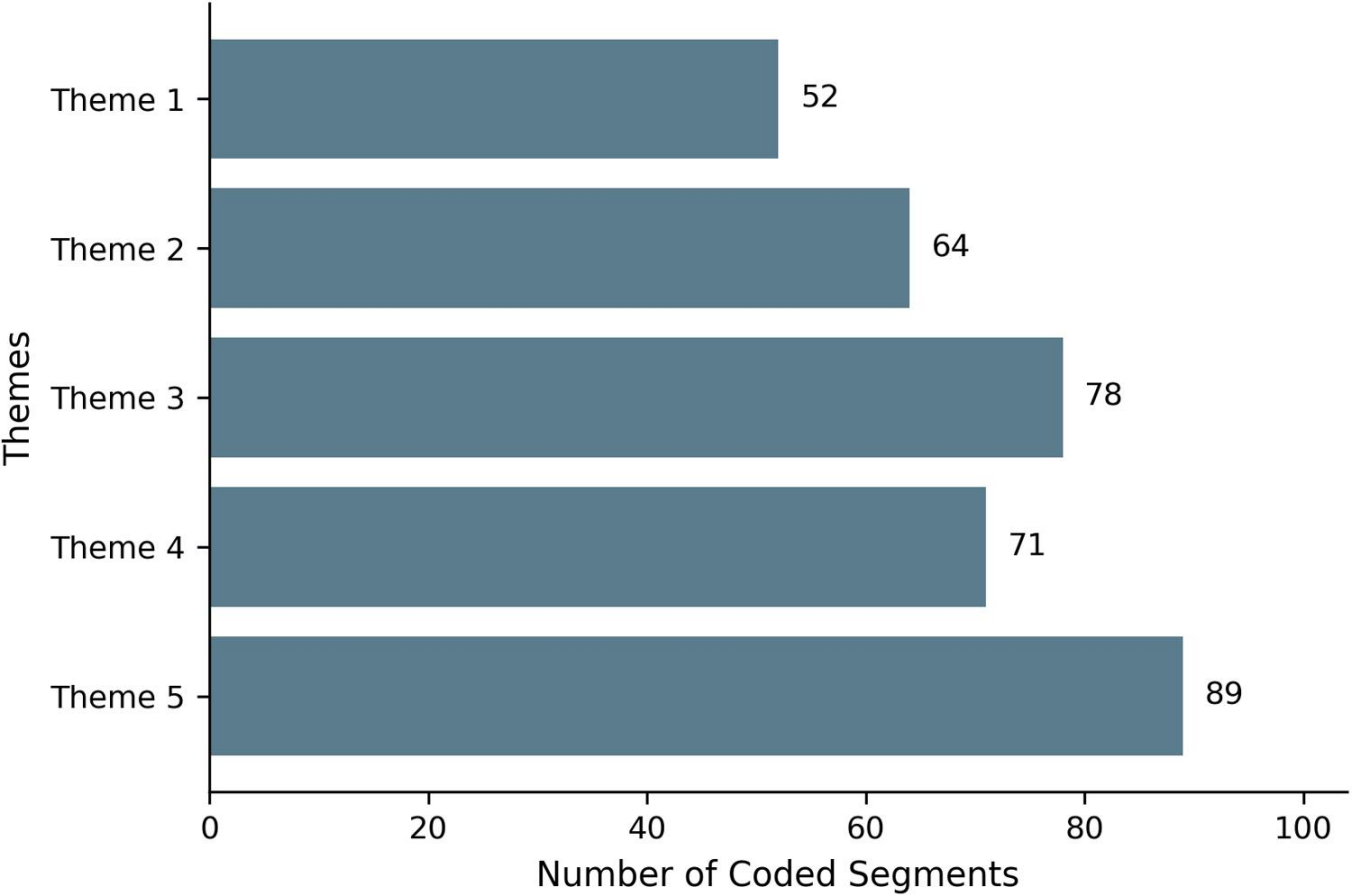
Distribution of coded segments across the five themes identified through reflexive thematic analysis (*N* = 354 segments). Counts reflect the relative analytic salience of each theme within teachers’ discussions and are provided to support transparency of the interpretative process rather than to suggest quantitative measurement.

To provide an overview of thematic density across the dataset, the number of coded segments contributing to each theme is presented in Figure 1. Responsible integration conditions (Theme 5) and structural constraints (Theme 3) represented the most densely coded areas, whereas conceptual ambiguity (Theme 1) was comparatively less prominent.

**Figure 2:**
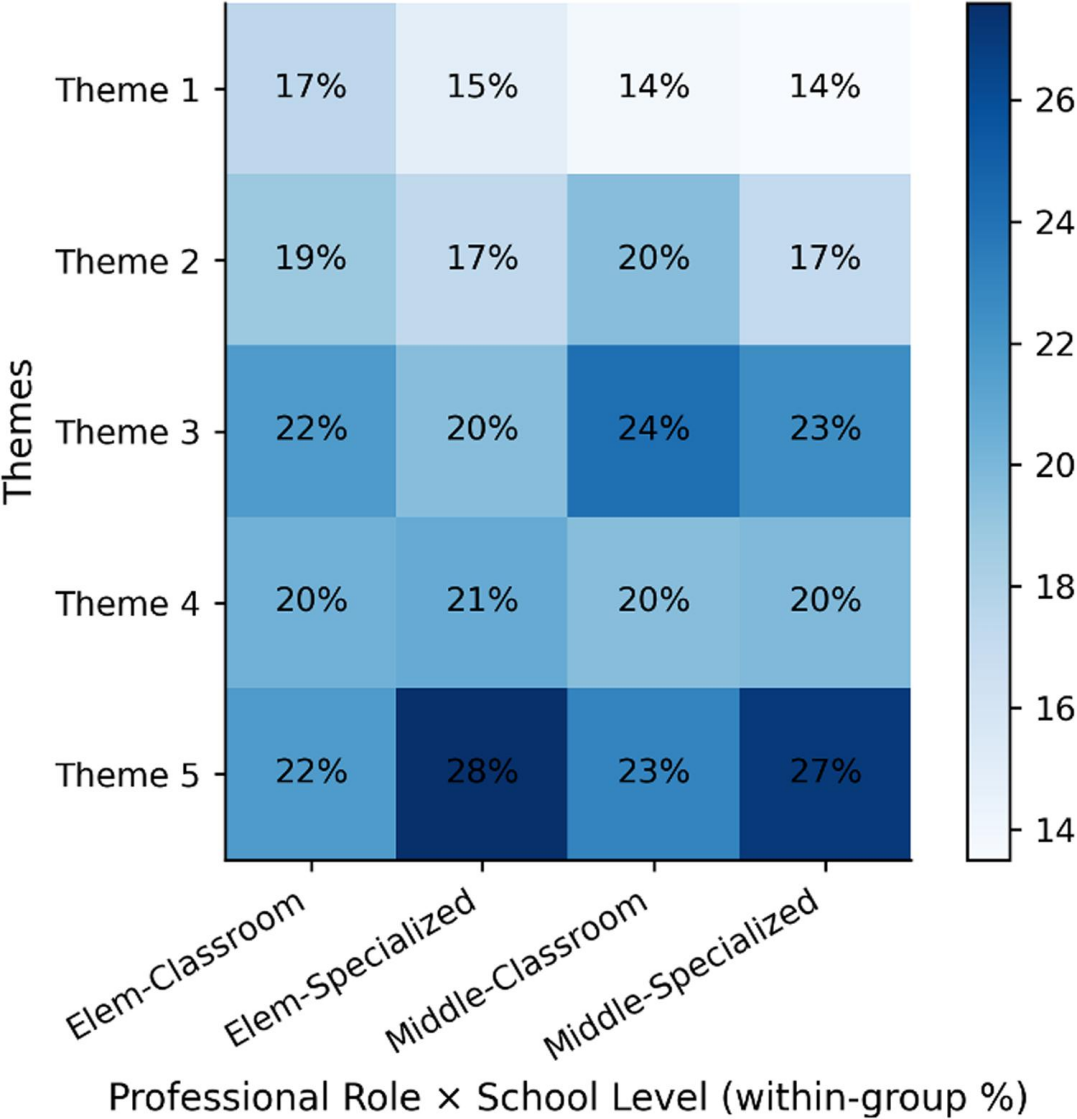
Thematic distribution across professional roles and school levels. Column-wise percentage distribution of coded segments within each subgroup. Each column sums to 100%, enabling comparison of relative thematic salience across roles and school levels.

To further examine whether thematic patterns differed across professional roles and school levels, a column-wise distribution of themes within each subgroup is presented in Figure 2. The underlying frequency counts supporting Figure 2 are reported in Supplementary Table S3. Across both professional roles and school levels, substantial convergence emerged regarding the conditional nature of technology integration. Teachers consistently emphasized professional judgment, human oversight, and the need for institutional support as foundational conditions for responsible implementation.

At the same time, some nuanced variations were observed. Specialized teachers tended to articulate slightly more elaborated reflections on personalization and ethical safeguards, particularly in relation to data sensitivity and potential stigmatization, whereas classroom teachers more frequently framed adoption in terms of feasibility within everyday instructional constraints.

Differences across school levels appeared subtle. Elementary teachers more often foregrounded relational and developmental considerations, whereas middle school teachers more frequently referenced policy alignment and curricular integration. Importantly, these variations did not alter the underlying convergence around professional agency as the central mediating factor.

## 4 Discussions

This study examined how teachers in inclusive educational settings serving students with neurodevelopmental differences conceptualize, evaluate, and regulate digital AT and emerging AI-based systems. Across focus groups, teachers described a logic of conditional integration: technologies were not seen as inherently inclusive, but as pedagogical resources whose legitimacy depended on contextual fit, professional control, and institutional feasibility. While traditional assistive technologies were typically framed as concrete supports for student autonomy embedded in established practices, AI-based systems elicited greater conceptual uncertainty and were more strongly associated with governance and ethical considerations.

### 4.1 Conditional integration, governance, and professional agency in neurodivergent contexts

**Figure 3:**
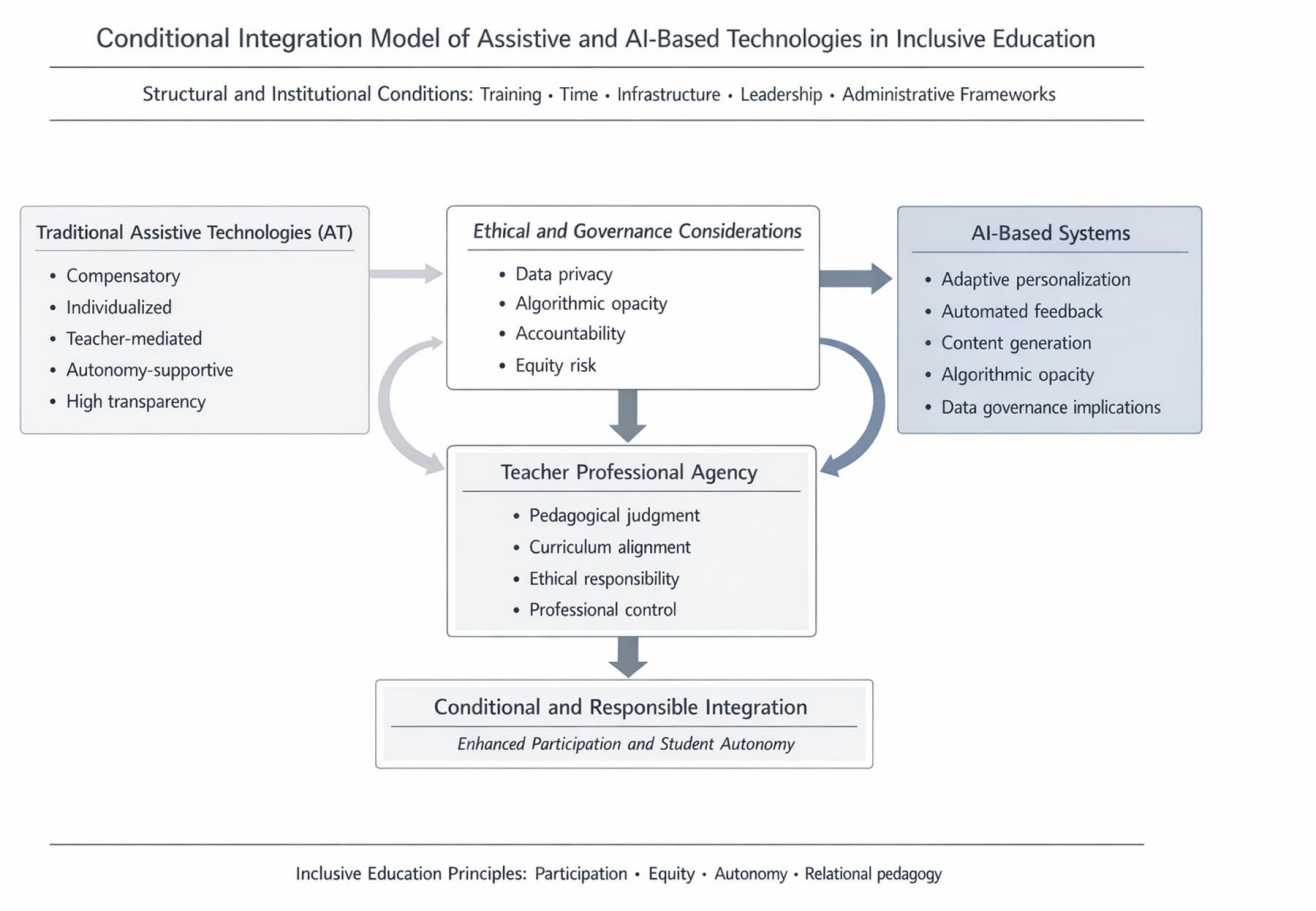
Conditional integration framework of traditional ATs and AI-based systems in inclusive education.

The five themes converge into a conditional integration framework (Figure 3) in which technology use is understood as mediated by the dynamic interaction between professional agency and structural conditions. Within this framework, technological legitimacy does not derive from innovation *per se*, but from contextual alignment, the preservation of pedagogical control, and institutional feasibility. Integration thus emerges as a negotiated accomplishment rather than a linear process of adoption.

Traditional AT were largely framed within individualized and compensatory logics, often tied to formal identification and targeted support needs. However, interactional exchanges revealed that this framing is negotiable: some participants repositioned assistive tools as resources for broader participation, indicating that the boundary between individualized accommodation and more universal use remains fluid in practice. These negotiations illustrate how teachers’ knowledge and interpretative work shape whether technologies become embedded supports or remain narrowly applied interventions.

AI-based systems were interpreted as qualitatively different because they mediate instructional processes through adaptation, recommendation, and content generation. Acceptance hinged on how control was distributed. AI was more readily accepted when positioned as a teacher-facing support (e.g., lesson preparation, material adaptation) than when imagined as directly guiding student learning. In this sense, professional agency extended beyond selecting tools to overseeing algorithmic outputs, assessing their appropriateness and alignment with pedagogical intent (20). Technologies were accepted not when they automated decision-making, but when teachers retained oversight and final judgment. This aligns with longstanding arguments that teacher judgment is central to responsible educational practice (41).

At the same time, integration was consistently described as structurally constrained. Limited training, time pressure, infrastructural instability, and administrative demands shaped adoption beyond individual attitudes. Integration therefore emerged as a socio-technical accomplishment, dependent as much on organizational coherence as on professional agency.

Ethical reflection became most pronounced in relation to AI-based systems. Concerns regarding data privacy, algorithmic opacity, accountability, and equity were articulated as practical dilemmas rather than abstract risks. Teachers questioned how to justify AI-mediated decisions to families, safeguard sensitive student information, and remain accountable when algorithmic reasoning was not fully transparent (42). Importantly, AI was not rejected categorically; its viability was framed as contingent upon maintained pedagogical judgment, transparency, and human oversight. These concerns resonate with emerging international frameworks for the governance of AI in education. For example, UNESCO (43) and OECD (44) emphasize principles such as human oversight, transparency, accountability, and data protection as foundational conditions for responsible AI adoption in educational systems. Teachers’ reflections in this study closely align with these governance principles: participants consistently emphasized the need to retain professional judgment, ensure explainability of algorithmic outputs, and clarify institutional responsibility for data management. From this perspective, classroom-level practices can be understood as the operational site where broader governance frameworks are translated into everyday professional decisions. The findings therefore suggest that responsible AI integration in inclusive education depends not only on regulatory guidelines but also on teachers’ capacity to enact these principles through situated pedagogical judgment.

These concerns are particularly consequential in neurodivergent contexts, where heterogeneous support needs and individualized trajectories heighten the consequences of misaligned recommendations (28). Teachers’ insistence on retaining professional control suggests that responsible innovation in inclusive settings depends less on technological expansion and more on safeguarding relational and pedagogical work. These findings extend the implementation literature by showing that the underexplored interpretative layer is not merely attitudinal but centers on ongoing negotiations around legitimacy, responsibility, and control, particularly when technologies redistribute decision-making authority.

Although subgroup differences were not a primary analytic focus, the descriptive patterns observed across roles and school levels are theoretically meaningful and emerged across several thematic discussions. Specialized teachers appeared somewhat more oriented toward personalization logics and the safeguarding of sensitive student needs, whereas classroom teachers more often foregrounded feasibility within whole-class instruction, curriculum pacing, and workload. Similarly, elementary teachers tended to emphasize relational and developmental dimensions of technology use, while middle school teachers more frequently situated technology integration within subject-specific demands, assessment pressures, and institutional accountability. These variations suggest that teachers’ interpretations of assistive and AI-based systems are shaped not only by individual attitudes, but also by the pedagogical responsibilities and organizational expectations associated with their professional context. Rather than indicating fundamentally different positions toward technology, these differences appear to reflect how distinct professional roles and school environments shape the practical conditions under which digital tools are evaluated and integrated.

### 4.2 Implications for policy, professional development, and design

Three implications for policy, professional development, and design follow from these findings.

#### 4.2.1 Professional development

Teachers’ uncertainty around AI, often negotiated through peer discussion, points to the need for AI literacy embedded within inclusive pedagogy, rather than standalone technical training. Professional learning should explicitly support teachers in distinguishing between traditional assistive tools and AI-mediated systems, with attention to how each redistributes control, introduces new accountability demands, and affects inclusive practices. Training should also be role-sensitive, recognizing that classroom and specialized teachers may prioritize different feasibility constraints and ethical safeguards (26). In particular, AI literacy should not focus solely on functional use, but on developing teachers’ capacity to critically evaluate algorithmic outputs, assess contextual appropriateness, and articulate decision rationales to students and families (20).

#### 4.2.2 Policy and governance

Teachers’ concerns regarding accountability and data governance indicate that implementation frameworks should clearly specify responsibilities related to data management, platform procurement, and the justification of AI-mediated decisions (43, 44). Governance should not be framed solely as regulatory compliance, but as an institutional architecture that enables informed professional discretion and clarifies how data, decisions, and responsibilities are distributed across stakeholders. Including teachers’ voices in institutional policy-making could strengthen the pedagogical legitimacy of technological choices, reduce operational uncertainty, and support coherent practices across classrooms and professional roles.

#### 4.2.3 Design

Design priorities should extend beyond usability to include explainability, controllability, and transparency, given well-documented ethical concerns in AI in education (42). Teachers’ discussions indicate that AI systems are perceived as more viable when they offer interpretable rationales, allow educators to adjust or override recommendations, and generate documentation compatible with existing accountability practices. At the same time, designers should account for infrastructural variability and unequal resource distribution, as AI-enabled tools risk reinforcing existing educational inequities if implemented without attention to systemic conditions (45).

#### 4.2.4 Classroom implementation

Beyond policy and professional development, the findings also suggest several practical considerations for classroom-level implementation. First, AI-based tools appear most pedagogically acceptable when they function as teacher-mediated supports rather than autonomous instructional agents. Teachers in this study described using AI primarily for preparatory tasks such as adapting texts, generating differentiated materials, or supporting lesson planning. Second, responsible classroom integration requires maintaining clear pedagogical oversight: algorithmic suggestions should be treated as provisional inputs that are evaluated and contextualized through teacher judgment. Third, inclusive implementation benefits from collective use strategies, where assistive or adaptive tools can be integrated into whole-class activities rather than being restricted to individualized remediation, thereby reducing stigmatization and supporting broader participation. Finally, teachers’ discussions indicate that transparent communication with students and families about how digital and AI-based tools are used in learning processes may help reinforce trust and accountability. Taken together, these practices illustrate how governance principles and ethical safeguards can be operationalized within everyday classroom decision-making.

### 4.3 Limitations and future research

This study is limited by its regional focus (Italian-speaking Switzerland and Northern Italy) and by relying on self-reported perspectives derived from focus group discussions rather than direct observation of classroom practice. Moreover, future research could integrate observational approaches or *in-situ* classroom discussions to examine how teachers’ interpretations translate into enacted pedagogical practices. Although descriptive subgroup patterns were explored, the study was not designed for systematic comparisons across roles, school levels, or experience with AI tools. The group-based format may also have influenced how positions were expressed through interactional dynamics. The study also reflects teachers who voluntarily participated, potentially indicating a higher level of engagement with technology-related issues. Future research should triangulate teacher perspectives with observational and longitudinal designs to examine how conditional integration evolves over time, how governance policies shape practice, and how specific AI-enabled tools are enacted in real inclusive classrooms. Given the rapidly evolving AI landscape, continued qualitative inquiry is needed to examine how teachers’ conceptualizations evolve as tools become more embedded in educational systems.

The findings of this study suggest that implementation operates as a process of conditional integration, in which technologies acquire legitimacy only when aligned with professional control, institutional feasibility, and ethical accountability. In neurodivergent contexts, sustainable integration depends less on technological expansion and more on how teachers exercise professional judgment over AI-based systems.

## Data Availability

The raw data supporting the conclusions of this article will be made available by the authors, without undue reservation.
