# Supplementary material for: Negotiating assistive technologies and AI in inclusive education: professional agency in neurodivergent contexts
Source: Front Child Adolesc Psychiatry. 2026 Apr 13;5:1820276. doi: 10.3389/frcha.2026.1820276 (PMC13111448; doi:10.3389/frcha.2026.1820276)
Supplement: Supplementary file 1 [file Datasheet1.pdf]

Negotiating Assistive Technologies and AI in Inclusive Education: Professional Agency in Neurodivergent Contexts

Supplementary Material

1 Supplementary Tables

Table S1. Semi-Structured Focus Group Discussion Guide

Section	Core Question	Follow-Up Probes / Prompts
**1. Inclusive Education Context**	How would you describe inclusive education in your daily professional practice?	What does inclusion look like in your classroom? How do you support students with neurodevelopmental differences?
**2. Understanding of Assistive Technologies**	When you hear the term "digital assistive technology," what comes to mind?	Can you provide examples? Who are these tools typically intended for? Are they used individually or with the whole class?
**3. Artificial Intelligence in Education**	What does "artificial intelligence" in education mean to you?	How is AI different from other digital tools? Have you encountered AI-based systems? In what contexts?
**4. Perceived Pedagogical Value**	In your experience, when are digital or AI-based tools useful in inclusive educational settings?	For which students? For which activities? Do they support autonomy, participation, personalization?
**5. Adoption Decisions**	What influences your decision to adopt or not adopt a digital or AI-based tool?	Training? Time? Infrastructure? School leadership? Evidence of effectiveness? Compatibility with curriculum?
**6. Professional Role**	Does your professional role influence how you evaluate or use these tools?	Differences between classroom and specialized teachers? Collaboration with colleagues?
**7. Ethical Considerations**	Are there any risks or concerns you associate with AI-based tools in inclusive education?	Data privacy? Transparency? Dependency? Equity? Professional responsibility?
**8. Implementation Conditions**	What would need to be in place for you to feel confident integrating these technologies into practice?	Professional development? Institutional guidelines? Technical support? Human oversight?

**9. Closing Reflection**	Is there anything else you would like to add about digital or AI-based tools in inclusive education?	—

Note. The discussion guide was used flexibly. Questions were adapted to the flow of discussion, and additional probes were introduced to clarify emerging topics or explore points of agreement and divergence within the group.

Table S2. Illustrative Open Codes and Their Development Into Subthemes and Themes

Illustrative Open Codes (Generated Across Focus Groups)	Subtheme	Final Theme
"AT is for students with certification"	Individualized compensatory framing	**Theme 1: Ambiguity and Divergent Conceptualizations**
"Tool for one specific student"	Deficit-oriented association	
"Whole class could use text-to-speech"	Universal or inclusive repositioning of AT	
"Not sure what counts as AI"	Conceptual ambiguity of AI	
"AI adapts automatically"	AI as adaptive system	
"AI makes decisions"	AI as autonomous or systemic agent	
"AI helps with lesson planning"	AI as professional support tool	
"Comparing examples across participants"	Collective negotiation of AI meaning	
"It must improve learning"	Pedagogical usefulness as adoption condition	**Theme 2: Conditional Acceptance and Contextualized Adoption**
"Needs to fit curriculum"	Curricular compatibility	
"Depends on school culture"	Institutional context as facilitator	
"Would like evidence first"	Evidence expectations	
"More careful with students using AI"	Differentiated acceptance (planning vs student use)	
"No proper training"	Training gaps	**Theme 3: Structural and Organizational Frictions**
"No time to experiment"	Time constraints	
"Internet doesn't work"	Infrastructure reliability	
"Too much paperwork"	Administrative burden	
"Need coordination between roles"	Organizational alignment	
"Where does the data go?"	Data privacy concerns	**Theme 4: Ethical Tensions and Professional Responsibility**
"I cannot explain how it works"	Algorithmic opacity	

"Students might rely too much"	Dependency risks	
"Could increase gaps between schools"	Inequality amplification	
"If we monitor it, it could help"	Conditional ethical optimism	
"Others agreed and emphasized oversight"	Collective reinforcement of professional accountability	
"Need clear rules"	Governance and institutional guidance	**Theme 5: Preconditions for Responsible Integration**
"Need proper training, not just tools"	Sustained professional development	
"We should decide together"	Shared governance	
"Teacher must have final say"	Human oversight	
"Technology cannot replace relationships"	Relational core of inclusion	

Note. The table presents illustrative examples of open codes and their clustering into subthemes and themes. Codes were generated across the dataset and are not linked to specific participants or frequencies, consistent with a reflexive thematic analytic approach.

Table S3. Distribution of coded segments across professional roles and school levels.

Theme	Elem-Classroom	Elem-Specialized	Middle-Classroom	Middle-Specialized	Total
Theme 1	12	13	12	15	52
Theme 2	13	15	17	19	64
Theme 3	15	17	21	25	78
Theme 4	14	18	17	22	71
Theme 5	15	24	20	30	89
**Column total**	70	87	87	111	354
